# Can aid foster Africa's attainment of a just energy transition and external debt sustainability?

**DOI:** 10.1016/j.heliyon.2025.e42507

**Published:** 2025-02-06

**Authors:** Kalimanshi Nsakaza, Talumba Ireen Chilipaine

**Affiliations:** a United Nations World Food Programme, Zambia; b United Nations Economic Commission for Africa, Zambia

**Keywords:** Just energy transition, External debt, Official development assistance, Generalized methods of moments, Renewable energy, Foreign aid

## Abstract

This study evaluated the relationship between Aid and Africa's potential to achieve the Just Energy Transition and external debt sustainability. In contrast to the instrumental variables technique, the paper employed a 2-step system GMM to analyze data from 39 African countries between 2012 and 2022. The paper also used 7 indicators of access to energy and electricity on both renewable and fossil fuels to measure the Just Energy Transition (JET) and 8 indicators to assess the external debt landscape of the sampled countries. The net official development assistance received per capital was used to measure foreign aid. The findings indicate that, for Africa, there was a significant and positive relationship between the net official development assistance and the energy sector for renewable energy, but an insignificant positive relationship for fossil fuels overall. This suggests that, while Net Official Development Assistance (ODA) inflow to Africa increased with the use of renewable energy, it did not significantly change with the use of fossil fuels. The findings also demonstrated that while interest payments on long-term external debt had a negative impact on ODA to African countries, the total external debt stock, mainly for Africa, had a positive and significant effect on the Net ODA received by the continent. The study concluded that in driving the JET, solar and bioenergy energy are a crucial renewable energy source for Africa that have the potential to revitalize the continent's renewable energy market as it. We recommend that Power market frameworks are a viable alternative that can be created by policymakers to promote and draw in private sector investment. Through innovative financing methods including securitization, blended finance, or public-private partnerships, the private sector can also use its knowledge of financial de-risking. For example, bonds backed by the cash flows from future renewable energy assets can be issued through securitization.

## 1 Introduction

Economic development cannot be achieved with a faltering energy sector, yet over 600 million people on the African continent continuously face daunting challenges in accessing such integral part of economic activity. Exorbitant and inflationary energy prices, as well as poor service quality, continue to limit consumption across Africa. The continent also faces significant obstacles in changing its social-economic dynamics due to unsustainable external debt and the energy crisis as 30 nations currently experience regular power outages, with many citizens paying enormous costs for backup power options. Given Africa's comparatively new energy infrastructure developments and the declining cost of green energy technologies, the continent is presented with a lucrative chance to rapidly transition to clean energy alternative solutions. The region's abundant clean energy resources may thus be a viable solution to the coupled dilemma of unsustainable debt and energy crisis [1]. However, it comes at a great cost [2,3], particularly due to Africa's limited revenue streams which possess a significant strain on both the state and household expenditure patterns. As a result, international aid, particularly in the form of official development assistance undoubtedly plays a vital part in financing and distributing clean energy technologies that unlock new avenues for resources pertinent to curb the continent's external debt burden [[4], [5], [6], [7]]. From 2000 to 2018, Africa's official development assistance for the energy sector increased from 1.9 billion USD to an excess of 11 billion USD.

Africa's aggregate financing requirements for adapting appropriately to climate change through the Just energy transition was estimated at about 1.3 trillion USD and 1.6 trillion USD according to the [8]. Adaptation costs are solely anticipated to surge approximately 407 billion USD in the next two decades. Over the same time period, the total amount of adaptation money required from global funds is expected to be 4.5–7 billion USD for the energy sector [9]. East Africa has the largest anticipated cost for adaptation initiatives (91–143 billion USD) owing to its increased sensitivity to climate vulnerabilities and also calls for the greatest commitment from international resources (58–92 billion USD) to achieve its adaptation demands. Central Africa on the other hand faces the lowest estimates of about 6–19 billion USD followed by West Africa (74–116 billion USD), North Africa (34–53 billion USD), and Southern Africa (25–42 billion USD) [9]. On the other hand, the continent continues to face the issue of unsustainable external debt, which has been compounded by the recent change in the trend of Official Development Assistance (ODA) being delivered through loans as opposed to grant financing [10]. states that from 2021 to 2022, ODA grants to developing countries, including Africa, fell by 8 percent to 109 billion USD, while loans rose by 11 percent to 61 billion USD, indicating a lower degree of concessionality in development assistance, with equity investment accounting for only a small portion of the total, which fell by 12 percent to 1.5 billion USD. The proportion of ODA grants surged in 2006 due to significant debt relief provided through the Heavily Indebted Poor Countries initiative and the accompanying Multilateral Debt Relief Initiative. From then, it has continued to be steadily declining. Between 2012 and 2021, ODA funding accounted for an average of 68 percent of the total and by 2022 plummeted to 63 percent.

In light of this, there has been a general consensus in the literature on the significance of aid, particularly ODA, in reorienting Africa's green energy sector and reducing the continent's soaring external debt. In order to achieve this dual objective, ODA's primary goal in Africa is to sufficiently support renewable energy initiatives that progressively replace the use of coal, oil and biomass systems, as well as to finance sturdy infrastructural development that unlocks new domestic resource avenues, thereby further reducing the need for external debt. As many African national institutions are unstable, the strength of these ODA recipient countries' internal institutions is important to its performance [11]. The underlying constraints behind the backlog in realizing ODA's impact on the Just Energy Transition (JET) in Africa has been due to the continent's widespread lack of institutional transparency which further fosters corruption [12]. For aid providing countries, this full disclosure of the ODA beneficiary country's national institutional set-up is both a requirement to conduct successful ODA flow monitoring and credit approval for Development Assistance Committee (DAC) member countries to engage in subsequent phases of most ODA initiatives [13]. An opaque system substantially decreases the positive impact of ODA for recipient nations, primarily owing to the disproportionate effect of aid resources, as each ODA financed project has a predetermined proportion of aid resource allocation, and if the project's execution diverges from the ideal ratios, the final effect is significantly hampered. It is additionally complicated to track the achievement of aid objectives hence, a transparent working institutional framework in the recipient country is an assurance of effective oversight and is closely tied to the level of due diligence demonstrated by the recipient's authorities. However, sustainable growth in Africa has not met ODA's commitments, and in certain countries, ODA is viewed as a source of market turmoil [14].

Debt sustainability and the JET thus necessitate that African nations exercise their potential in the rapidly developing global green-growth technology and renewable and clean markets. Africa's green mineral wealth creates unique prospects for the region to take the lead in an assortment of green development sectors, particularly green energy and electric vehicles battery technologies as over fifty percent of African countries possess at least one of the Critical Energy Transition Minerals (CETM) which are critical to the future global green economy. Africa is furthermore the highest quality continent for green energy deployment, accounting for more than 45 percent of the world's technical potential. However, revamping Africa's clean energy sector would necessitate technological improvements in order to balance economic expansion with natural resource conservation. Technological advancement alone will not be enough to move away from a traditional coal powered energy system; significant political determination, effective planning, and an integrated plan that fully utilizes green energy are also required. Although the unique roadways to a JET may differ based on the needs of different African countries, every avenue must be inclusive and equitable in order to contribute to the achievement of the United Nations Sustainable Development Goals [15]. A JET will improve the continent's economic development trajectory, enhance climate change resilience, and stimulate innovation to achieve sustainable development at all levels while attracting large investments.

The arguments we present in this paper are founded on the notion that proactively recognizing the linkage between aid in the form of ODA and the Just energy transition including external debt can result in considerably better outcomes than waiting too long for its effects to be realized under its current trajectory. Advancing knowledge in this aspect through achieving a fair and just energy transition will however necessitate a consensual strategy and process based on controversy and supported by a set of principles that guide it. The primary goal of this study is thus to inform African policymakers about the implications of strategic decision-making in external debt management and green energy development efforts through ODA which has largely been absent in the current context of the existing literature in this area.

## 2 Literature review

### 2.1 Official development assistance and external debt in Africa

For decades, the relationship between ODA and Africa's foreign debt burden has been a recurring and contentious topic in discussions about Africa's development predicaments and paradoxes. This is not surprising, considering its enormity and the implications for Africa. A number of academics, including [16], who attempted to explore the link between foreign aid, external debt sustainability, and economic growth in Africa, have stated that debt reduction does provide some opportunities for African development. At the very least, it marks a significant 'burden-lifting' in terms of servicing debt and investment withdrawal from Africa, which has hampered economic growth. As stated earlier in section 1.0, Africa's ODA trajectory has shifted significantly from grant to loan-based aid, which has hampered the continent's goal of achieving sustainable external debt levels.

In this paper, the argument we make is that the extent to which a country is dependent on foreign aid and vulnerable to external debt shocks is also determined by the significance of aid in relation to other external capital flows. Many African countries rely on ODA simply because they have restricted or no access to foreign capital markets and receive little FDI, the primary source of external capital for developing countries [17]. in contrast, proposed the introduction of moderately concessional loans (MCLs) in response to the concern about whether less concessional development assistance for Africa will result in an external debt crisis by stating that MCLs would be 40-year U.S. dollar priced loans at 3–4 percent interest (based on the United States 30-year Treasury bill) and 10 years of grace period. They concluded that moving to MCLs would not exacerbate debt sustainability issues in over half of the African countries because they would be less expensive and have a longer-term maturity than the current U.S. dollar Eurobonds, which have yields of 6–12 percent and generally have an amortization period of 10 years with an immediate repayment of the principal at maturity. In fact, they showed that if state resources were sufficiently augmented to replace costly market borrowing, the move to MCLs might actually strengthen debt sustainability.

### 2.2 Official development assistance (ODA) and the just energy transition (JET) in Africa

In recent academic studies, there has been a resurgence of interests for many scholars on the link between ODA and the JET [18,19]. The nature and extent of the causal relationship between ODA and JET, however, still remains a matter of debate among academic circles, as aid policy's importance is primarily determined by it. The most well-known form of aid is the ODA given to African nations by the Development Assistance Committee (DAC), which has three main goals; to lessen the effects of climate change; supply energy to local businesses founded by donor nations; and to meet the energy needs of the African continent. These objectives indicate that a cleaner, more dependable, and lower-carbon energy production paradigm is offered by renewable energy and this presents lucrative opportunities with the growth in renewable energy in Africa [20,21].

The goal of ODA for renewable energy in Africa is to raise the quantity of power produced using renewable resources. Since green energy can slow down the impacts of climate change and is essential to the successful execution of green energy projects, its rise is consistent with the objective of sustainable growth [22]. Some argue that aiding Africa to foster the smooth energy transition is a moral duty for wealthy nations and that doing so would significantly increase the region's renewable energy sources [23]. According to this school of thought, giving increases growth and yields more rewards [24]. despite this compelling argument, it however also makes sense to doubt the ODA's viability and effectiveness in light of the problematic implementation and utilization of ODA in many African nations over the last 60 years as a renewable energy source. Because of this, some have claimed that ODA should cease to be provided and that it has little effect on the growth of renewable energy [25]. ODA does not, in the eyes of donor nations, offer developing nations unconditional free financial support. Based on years of experience with foreign aid in Africa, donor countries' considerable subjective will is evident in ODA. With the ultimate goal of using assistance funds to support the adoption of laws and regulations that are more favorable to foreign investment, the DAC of the OECD is specifically more willing to assist recipient nations who have received greater investment from donor nations. This implies that ODA and foreign direct investment are essentially the same thing, and that ODA may merely be a fancy name for an old wine. Regardless of how extreme these opinions may sound, they have both brought forward valid points, necessitating a closer examination and comparison between ODA and attaining the JET in African nations.

In the Analysis of this relationship between ODA and the energy sector, it has been observed by some that there may be a non-linear relationship between ODA and JET achievement in African nations. The effectiveness of climate aid is significantly diminished when recipient nations take aid in a passive manner. These current studies are insufficient, despite the fact that they can offer abundant data for a thorough understanding of the link between the ODA and the advancement of renewable energy. Recent reports have suggested that there might be a threshold effect, that is, a non-linear relationship in which the model coefficients change dramatically as the value of the threshold parameter changes, between the ODA and the growth of green/renewable energy. This paper used the threshold effect model to test the nonlinear relationship. The difference between the two methods is that only when the relationship between the ODA and the renewable energy development changes from positive to negative, or from negative to positive, can the nonlinear relationship be tested. If the relationship is always positive or negative, previous studies cannot test the difference between the two, but the threshold effect model can verify the difference between the two.

Generally speaking, the impact of ODA on renewable energy development is not a single linear relationship [26,27]. Considering the potential influence mechanism between them, this study explored the threshold effect between them, that is, a nonlinear relationship. The purpose of understanding the nonlinear relationship is to verify whether the ODA really promotes the development of renewable energy. It is known that the ODA can influence the development of renewable energy by influencing energy structure and social structure. Our goal is to capture the influence of internal factors, through which the ODA may affect the development of renewable energy. Table 1 shows the summary statistics of the variables employed in the analysis with the total external debt exhibiting the largest variance relative to its mean while solar exhibited the lowest variance.

**Table 1:** Summary statistics.

Variable	Obs.	Mean	Std. Dev.
Net ODA per capita	429	70.63902	56.24228
Electricity generation (TWh)	429	11.2473	39.54829
Coal	429	6.027144	17.40524
Gas	429	11.82656	23.46806
Fossil fuel	429	55.32248	57.44738
Renewable	429	54.78733	126.9435
Solar	429	1.101729	2.141103
Bioenergy	429	2.352397	7.776052
Bilateral	429	1945.465	2251.819
Bondholders	429	3007.902	12167.77
Commercial Banks	429	4618.877	9719.968
Interest payments (Long term)	429	350.8135	916.2194
Long term external debt	429	11945.3	21714.43
Multilateral	429	2563.43	3136.589
Official Creditors	429	4522.048	4871.633
Private Creditors	429	4470.466	13178.64
Public sector external debt	429	9724.386	17743.01
Total External debt stock	429	15101.58	28738.66

*Note: Coal, Gas, Fossil Fuel, Solar, Renewable are all expressed in percentage of total electricity Production; External debt variables are expressed in million USD.* All data employed ranged from 2012 to 2022.

## 3 Data and measurability

### 3.1 Measurement of foreign aid

Prior to applying the methodology, it is important to define and give the measure of foreign aid. In its broadest sense, foreign aid refers to any resources given to recipient nations by donors, including financial grants (gifts), technical expertise, physical products, and gifts or concessional loans. Official development assistance (ODA) and official development finance (ODF) are the two main categories of foreign aid. ODA covers grants and concessional loans with a minimum 25 % grant component are covered by ODA and OA [28]. Both ODA and OA come from official sources and are primarily provided to support developing nations' economic growth and well-being. We employed the net official development assistance (ODA) received per capital in foreign aid. In order to support economic development and welfare in the countries on the Development Assistance Committee (DAC) list of ODA recipients, official agencies of DAC members, multilateral institutions, and non-DAC nations disburse loans and grants on concessional terms (net of principal repayments).

In addition, ODF is a subset of ODA. Non-concessional bilateral and multilateral development loans having a grant element of less than 25 percent are also included in the ODF [29]. Foreign aid can be classified into two broad groups, bilateral (two-sided) and multilateral (many-sided) aid. In the former case, aid is provided directly to a recipient government by a donor government. The latter is assistance provided by a global organization on behalf of several government funders. On occasion, though, a donor may enter into an agreement with a multilateral organization to have the agency carry out a project or program in a recipient nation. These situations are commonly referred to as Bi/Multi and are normally counted as bilateral flows. international organizations like the World Bank and the United Nations Development Programme (UNDP) that oversee multilateral aid. Furthermore, the donor nations' agencies oversee the administration of bilateral aid. Over two-thirds of all ODA from DAC member nations is given bilaterally, primarily in the form of grants, according to OECD data from 2009. A portion of the aid funding comes from for-profit entities such "non-governmental organizations" (NGOs).

### 3.2 Measurement of the just energy transition

In order to address the dual concerns of climate change and sustainability, the "Just Energy Transition" rhetoric places a focus on social justice in the energy transition [30]. "A long-term technological and socio-economic process of structural shift that affects the generation, distribution, storage, and use of energy and causes rearrangements at micro (innovation), meso (social networks, rules, and technical elements), and macro (exogenous environment) levels, while also ensuring that the desired socioeconomic functions can be accomplished through decarbonized and renewable means of energy production and consumption, safeguarding social justice, equity, and welfare" is the definition of a just energy transition, according to García-García et al. In ensuring robustness, we employed 12 indicators in measuring the JET namely; Electricity generation (TWh); Coal, Gas, Fossil fuel electricity per capita (kWh), Fossil fuel, Renewable, Solar, Bioenergy as a percentage of total energy produced; Renewable electricity per capita (kWh). This was also cardinal integrate a variety of clean energy technologies within the framework of the concept of “decarbonization” which is central to the JET and help re-orient Africa's definition of sustainability beyond Hydro sources.

### 3.3 Measurement of the external debt landscape

In measuring the external debt portfolio, we employed 11 variables and define the variables using the World Bank classification. The variables “Total External debt stock” measures the total external debt owed to nonresidents repayable in currency, goods, or services as total external debt is the sum of public, publicly guaranteed, and private nonguaranteed long-term debt, use of IMF credit, and short-term debt. “Bilateral” on the other hand measures Public and publicly guaranteed bilateral debt which includes loans from governments and their agencies (including central banks), loans from autonomous bodies, and direct loans from official export credit agencies. The variable “Bondholders” measures Public and publicly guaranteed debt from bonds that are either publicly issued or privately placed while “Commercial Banks” depicts the public and publicly guaranteed long-term commercial bank loans from private banks and other private financial institutions. “Disbursements” measures the disbursements on long-term debt that are drawings by the borrower on loan commitments during the year specified. Furthermore, “Long-term external debt” is defined as debt that has an original or extended maturity of more than one year and that is owed to nonresidents by residents of an economy and repayable in currency, goods, or services while “interest payments on long-term debt” are actual amounts of interest paid by the borrower in currency, goods, or services in the year specified.

The variable “Official Creditors” shows the Public and publicly guaranteed debt from official creditors which includes loans from international organizations (multilateral loans) and loans from governments (bilateral loans). Loans from international organization include loans and credits from the World Bank, regional development banks, and other multilateral and intergovernmental agencies with the exclusion of loans from funds administered by an international organization on behalf of a single donor government. “Private Creditors” includes Public and publicly guaranteed debt from private creditors including bonds that are either publicly issued or privately placed; commercial bank loans from private banks and other private financial institutions; and other private credits from manufacturers, exporters, and other suppliers of goods, and bank credits covered by a guarantee of an export credit agency. “Public sector external debt” comprises long-term external obligations of public debtors, including the national government of all levels, political subdivisions (or an agency of either), autonomous public bodies such as Public Corporations, State Owned Enterprises, Development Banks and Other Mixed Enterprises.

## 4 Empirical model

The purpose of this study was to assess the relationship between Foreign Aid in the form of ODA and the attainment of the Just Energy Transition and External Debt. Thus, the Mathematical Model can be expressed as;

ForeignAid=f(JustEnergyTransition;ExternalDebt)

As stated in section 3 that the measurement of a Just energy Transition required a number of indicators to ensure robustness, the variables representing the JET where thus 11 and comprised of;

JET=Electgeneration+Coal+Gas+Fossilfuel+Renewable+Solar+Bioenergy+Renewableelectricity

The measurement of the external debt landscape also required a number of indicators to ensure robustness and the variables representing the external debt where 12, comprising of;

ExternalDebt=Bilateral+Interestpayments+Longtermexternaldebt+Multilateral+OfficialCreditors+PrivateCreditors+Publicsectorexternaldebt+TotalExternaldebtstock

Combining both indicators, the empirical model can thus be re-written as;

ForeignAid=Electgeneration+Coal+Gas+Fossilfuel+Renewable+Solar+Bioenergy+Renewableelectricity+Bilateral+Interestpayments+Longtermexternaldebt+Multilateral+OfficialCreditors+PrivateCreditors+Publicsectorexternaldebt+TotalExternaldebtstock

We use the two step system GMM as employed by Ref. [31] but was first put forth by Ref. [32], and subsequently refined by Refs. [33,34]. The system GMM estimator has been suggested by Ref. [35] to significantly increase efficiency and circumvent the issue of weak instruments in the first-difference GMM estimator created by Ref. [32]. Firstly, first-order serial correlation, rather than second-order serial correlation, is required for the idiosyncratic error term in order to provide consistent estimation on the lagged dependent variable (the regressor). We limited the number of lags as instruments or collapsed the instrument set into a single column for all time periods in order to prevent the issue of instrument proliferation.

The system GMM is preferred in this study over the instrumental variables 2SLS approach as it accounts for the issue of reverse causality by employing the instrumental variables method with external instruments [36]. This method tends to neglect the endogeneity of other regressors, though, because it is rare to find a wholly exogenous external instrument that varies over time and from nation to nation. One benefit of the GMM is that it uses internal instruments to address the endogeneity of all the explanatory factors. Additionally, the GMM generates valid instruments and handles the endogeneity that would result from reverse causality. The standard IV estimator is a special case of a Generalized Method of Moments (GMM) estimator. The assumption that the instruments P are exogenous can be expressed as $E\left(P_{i}u_{i}\right)=0$. The M instruments give us a set of L moments,

$$q_{i}=P_{i}^{\prime }u_{i}=P_{i}^{\prime }\left(y-X_{i}\beta \right)$$

where $P_{i}$ is M × 1. The exogeneity of the instruments means that there are L moment conditions, or orthogonality conditions, that will be satisfied at the true value of $\beta =\beta_{0}$:

$$E\left(g_{i}\left(\beta \right)\right)=0$$

Each of the L moment equations correspond to a sample moment, and we write these L sample moments as;

$$q\left(\beta \right)=\frac{1}{n}\sum_{i=1}^{n}P_{i}^{i}\left(y-X_{i}\beta \right)$$

If the equation is overidentified, however, so that P > K, then we have more equations than we do unknowns, and in general it will not be possible to find a $\hat{\beta }$ that will set all L sample moment conditions to exactly zero. In this case, we take a P × P weighting matrix W and use it to construct a quadratic form in the moment conditions. This gives us the GMM objective function:

$$J\left(\beta \right)=n\bar{P}{\left(\beta \right)}^{\prime }WP\left(\beta \right)$$

Which thus yields the GMM estimator;

$$\beta_{GMM}={\left(X^{\prime }PWP^{\prime }X\right)}^{-1}X^{\prime }PWP^{\prime }y$$

As we estimate two Models with different set of instruments; our empirical models thus can be expressed as.

### 4.1 Model 1

X=Electricitygeneration+Coal+Fossilfuel+Renewable+Hydro+Solar+Bioenergy+Renewableelectricity

P=Bilateral+Multilateral+OfficialCreditors+PrivateCreditor+TotalExternaldebtstock

Note: K > P to avoid overidentifying the model.

### 4.2 Model 2

X=Bilateral+Bondholders+CommercialBanks+Disbursements+Interestpayments+Longtermexternaldebt+Multilateral+OfficialCreditors+PrivateCreditors+Publicsectorexternaldebt+TotalExternaldebtstock

P=Electricitygeneration+Fossilfuel+Renewable

Note: K > P to avoid overidentifying the model.

## 5 Results and discussions

### 5.1 Bivariate analysis

**Fig. 1:**
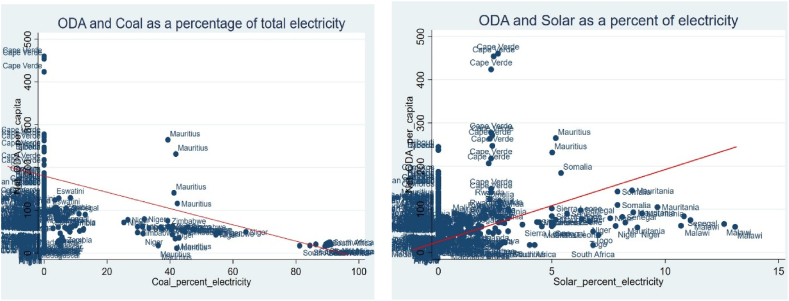
Oda and just energy transition.

Source: Authors computation based on data from the World Bank

Africa has been experiencing an acute shortage of energy in recent times, as its requirements continue to rise. Similar to many other developing and emerging continents, it has to choose between using readily available fossil fuels such as coal or making costly investments in renewable energy. By redefining ODA's function as an avenue to raise external finance for the continent's energy sector in support of JET, Fig. 1 informs the present global discussion on the Green Economy in context.

The Bivariate analysis presented in Fig. 1 shows that the relationship between ODA and green energy (solar) as a percentage of total energy production was positive while that of fossil fuels (coal) was negative, emphasizing the significance of ODA in aiding the transformative agenda of African countries towards a just energy transition. For Africa however, ODA has been seen to flow more to fossil energy industries like the coal sector in the long run than to the growth of the renewable power industry, which is likely to increase environmental damage in the long run [37]. Although a consensus on the relationship between ODA and the energy sector in recipient African nations has been difficult to reach [38], however found that ODA recipient countries ought to utilize large-scale green aid initiatives in order to attain focused energy sources, which can subsequently lead to a reduction of their dependence on fossil fuels. To enhance the capacity of ODA in fostering the JET, a number of initiatives in Africa have been developed to anchor in investment in the renewable energy sector through ODA including the Green Guarantee Company (GGC) which is an which is an institution that offers guarantees to institutional investors purchasing green bonds issued and listed on the London Stock Exchange and green loans issued in the private credit market, and has an aim of mobilizing billions of dollars in climate finance for developing nations and obtains funding from the Nigeria Sovereign Investment Authority, Green Climate Fund, the United States Agency for International Development (USAID) in partnership with Prosper Africa, Norfund, and the Foreign Commonwealth & Development Office of the United Kingdom through its MOBILIST program.

The basic argument has however been that while ODA and the energy transition exhibit a positive relationship, if Africa is going to Fund the energy transition from ODA, there is need to double it. On one side, the expenses of assisting developing African nations attain a Just energy transition may just exceed aid's ability to accomplish much at all, as it has never been able to address all of the issues it is already tasked with. According to United Nations Framework Convention on Climate Change (UNFCCC) biennial reports, ODA makes up approximately 84 % of bilateral climate money. The percentage of outflows from ODA-eligible multilaterals with a climate focus rose from 15 % to 28 % between 2013 and 2018. This implies that between 2013 and 2018, ODA accounted for over $15 billion USD in additional climate finance. However, over the same time period, the total gross ODA for comparable nations increased by just $12 billion USD. Additionally, throughout this time, the costs incurred by in-country donors to house refugees have increased by $6 billion USD. This implies that between 2013 and 2018, the amount of financial assistance actually utilized in developing nations for non-climate-related initiatives decreased by almost $9 billion USD. If developed nations insist on using ODA to finance the Just Energy Transition, then ODA must be increased at a rate never experienced before. This would imply that the US would spend 60 billion USD on a proportionate increase, France would spend just under 1 percent of GNI, and Germany, the Netherlands, and the UK would each spend well over 1 percent of GNI in order to meet current commitments to mitigation alone, without further cutting existing funds for development. It is necessary to look beyond ODA for climate finance if these figures don't appear realistic [39].

### 5.2 Does the nature of external debt matter?

Although African nations have seen a considerable increase in financial resources from both bilateral and multilateral creditors, which has allowed them to increase their import capacity, these nations have not been able to solve their crises including debt-overhangs or lower their total level of indebtedness due to the growing share of pure grants and debt relief from bilateral donors in recent years.

**Fig. 2:**
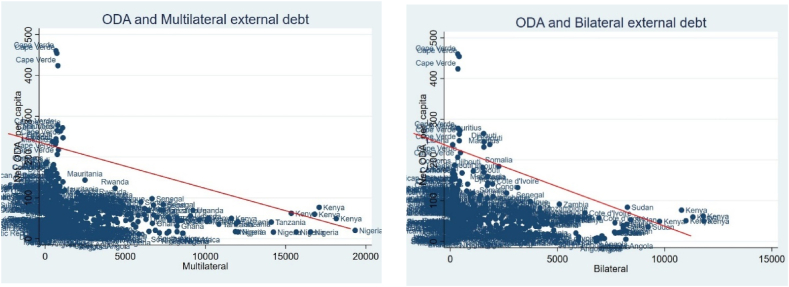
ODA and the type of external debt.

Source: Authors computation based on data from the World Bank

Fig. 2 indicates that the net official development assistance (ODA) received by recipient African countries was negatively correlated with external debt, regardless of the type of creditors. This is intriguing because African sovereigns that have trouble repaying their debts are more likely to default on official bilateral creditors through missing payments more frequently and in larger amounts than to multilateral creditors or bondholders. Therefore, even though bilateral lending appears to have a negative impact on ODA, it actually implies smaller cash flows because it is "pseudo" or "accounting" money and because it leads to a reduction in new lending from bilateral sources. Additionally, this crowding-out effect between ODA and new lending from bilateral sources shows that bilateral lenders are moving away from concessional and non-concessional lending to a mix of pure grants and debt reduction from the Official Development Assistance (ODA) for Africa.

ODA grants to developing countries fell by 8 % to $109 billion between 2021 and 2022, but loans rose by 11 % to $61 billion, suggesting that development assistance was less concessional. Only a minor portion of the total is made up of equity investment, which dropped by 12 % to $1.5 billion. At a time when many developing nations are still fighting to regain fiscal flexibility following the COVID-19 pandemic and to deal with the consequences of global monetary tightening, there is an increasing reliance on debt-creating ODA instruments. This makes debt sustainability worse, particularly for high-risk or distressed recipient nations. The International Development Association, the World Bank's concessional lending arm, have consistently expressed concerns that a large shift to debt would make it challenging to keep debt repayment and compliance from debt-stricken African countries at the current level. They worry that a change of this kind would reduce public support for aid to developing nations in donor nations. One question is unfortunately commonly disregarded in this debate. Will recipient nations' finances be impacted by the anticipated switch from grants to loans for foreign aid? Budget planning may become more challenging and aid dependency may increase if bigger loans result in lower domestic income.

This situation could occur in a number of ways including; First, compared to tax revenues, aid is far more erratic and unpredictable, and in nations that rely heavily on aid, volatility is a bigger issue. Additionally, research has demonstrated that loan help is more erratic than grant aid. Second, nations may start depending on aid to fund their efforts to combat poverty, which would have to be reduced if aid inflows decreased or stopped. Third, governments may be less motivated to maintain effective institutions and enact sound policies if they become increasingly reliant on help. Fourth, broad tax exemptions for powerful interest groups and poor tax compliance result in low tax revenues in many countries; hence, increasing assistance inflows could take focus away from fixing these governance flaws. Fifth, there is some indication that fiscal changes based on a stronger revenue effort are more sustainable in low-income nations. Therefore, nations may be less determined to pursue fiscal consolidation if they receive greater loans.

### 5.3 System GMM results

Table 2's results indicate that, for Africa, the relationship between net official development assistance (ODA) and the energy sector was insignificant and positive for fossil fuels but positive and significant overall for renewable energy. This suggests that, while net ODA inflow to Africa increased with the use of fossil fuels, ODA decreased with the use of renewable energy. The self-sustaining mechanism of this relationship makes the fossil fuel energy sector more direction-dependent and may hinder the development of the just developing African energy industry that is focused on accomplishing the Just energy Transition. If this is the case, then it is reasonable to assume that implementing the holistic transition pathway will only temporarily increase the amount of renewable energy used. This will intensify the current trend of ODA flowing more into the conventional energy sector powered by fossil fuels than into new green energy resources, thereby impeding the continent's goal of zero net emissions.

**Table 2:** ODA and the just energy transition.

Variables	(1)	(2)	(3)	(4)
Net ODA per capita (L1)	0.0474	0.4731^a^	0.2194^a^	0.3924^a^
	(0.0538)	(0.0601)	(0.078)	(0.1127)
Fossil fuels	0.1918	0.2702	−0.5279	−0.0744
	(0.3911)	(0.2222)	(0.5254)	(0.7712)
Renewable Energy	0.0364^a^	−0.1262	0.1346^a^	−0.0766
	(0.0098)	(0.1689)	(0.0084)	(0.3182)
Coal Energy	−2.6582^c^	0.3718	−3.0585	2.8176
	(1.5424)	(0.7411)	(1.9179)	(4.5709)
Solar Energy	0.1179	0.3984^a^	3.3381	16.914^a^
	(0.0714)	(0.0015)	(3.5389)	(6.5246)
Bioenergy	−0.7517^a^	0.2791∗∗	−0.4926	2.4181
	(0.0883)	(0.1233)	(2.1273)	(3.2371)
Gas Energy	−2.9938^b^	−0.6924	−0.9776	−1.1101
	(1.2482)	(0.9471)	(0.7863)	(1.4201)
Electricity generation	−1.3051	−2.6482	−3.9477^b^	−3.8928^b^
	(2.5867)	(2.1212)	(1.7576)	(1.2674)
Constant	130.8857^a^	72.3317^a^	157.64^a^	81.8481
	(27.6945)	(17.9381)	(39.6801)	(61.7408)
AR(1)	−1.39	−1.41	−1.77	−1.81
AR(2)	−1	−0.82	−1.24	−0.47
Sargan OIR	12.79	17.11	6.22	5.55
Hansen OIR	11.74	16.14	15.04	12.92
Instruments	21	21	21	21
Countries	39	39	39	39
Observations	390	390	390	390

a = P < 0.001.

b = P < 0.05.

c = P < 0.1.

If aid continues to accentuate their existence, this will also make the transition from the current energy structure to renewable energy more challenging. Solar energy, which according to baseline results had a positive and significant effect on ODA at all lags (1–4), is a crucial renewable energy source for Africa that appears to have the potential to revitalize the continent's renewable energy sector. That said, Africa's solar energy industry is still in its infancy compared to other countries; the continent's grid capacity is barely 90 MW, and solar electricity is concentrated in rural areas rather than cities [38]. Because solar energy provides a steady and reliable power, the mining industry is one of the biggest users of these energy sources. However, it has been observed that donor nations' ability to actively develop renewable energy in urban areas, where most industrial production typically takes place, is compromised when they develop solar grids supply in remote rural areas in accordance with their own interests when awarding ODA, which prevents the combined effect of urban solar development initiatives from being achieved.

The results also show a positive relationship between ODA and Renewable energy sector including Bio-energy in Africa. The high productivity of biomass, particularly from agricultural commodities including sugar cane, sorghum, and cassava, as well as from wood and agricultural waste, presents enormous prospects for modern bio-energy in Africa. In Africa, non-edible oils like jatropha oil have recently been identified to be viable feedstock for biodiesel. Jatropha may thrive in a broad range of land and climate conditions. In terms of photosynthetic efficiency, African nations are five times more productive which thus presents Africa countries with the potential for bioenergy of any place in the world due to the region's vast tracts of suitable crop and grazing land, ideal climate, and inexpensive labor.

Africa has enormous potential for producing bioenergy, but this potential has not been fulfilled due to a lack of cooperation between national and local authorities, insufficient institutional frameworks, and an inability to create and execute suitable policies. A critical issue that arises with the expansion of Bioenergy is that use of forest areas must increase in order to expand bio-energy. production Deforestation and the destruction of natural ecosystems and landscapes are being caused by large-scale, monocrop bioenergy crop plantations that are being developed at the expense of natural forests. The main worry is that it is hard to locate undeveloped area without affecting food production and vital ecosystems, including natural forests. Due to competition for land, water, and crops as feedstock, there are worries that the development of biofuel feedstock may replace the production of food crops and have a detrimental impact on prices.

In order to create an environment that encourages investment in renewable energy in Africa, policies and laws are essential components. Consistently establishing national roadmaps, goals, and objectives communicates a clear message to investors and the private sector about Africa's long-term future. These plans should be created and carried out after extensive stakeholder participation. They should contain attainable goals and opportunities for implementation through legislation and regulations that give businesses a secure investment climate. Morocco, for example, has set a lofty goal to have 52 % of its energy come from renewable sources by 2030. In order to accomplish this goal, the North African nation has created a long-term national energy strategy that details the laws and policies required. The creation of renewable energy zones and the creation of a renewable energy fund to encourage project investment are two components of this plan. Increased appeal to private sector businesses can also be attained by putting regulations like feed-in tariffs, net metering, and tax incentives into place. For instance, the government of South Africa established the Renewable Energy Independent Power Producer Procurement Program in 2011. This program offers renewable energy companies long-term power purchase agreements, creating a secure investment climate for the industry.

Furthermore, Kenya which is widely considered to be at the forefront of Africa's development of renewable energy also plans by 2030 to have all of its energy come from renewable sources. The nation's National Energy Policy, which was introduced in 2019, details this audacious objective including several initiatives the country has put in place to accomplish this goal, such as updating its feed-in tariff, instituting net metering, offering tax breaks, and implementing additional financial support systems [40]. Among other things, the Kenyan government's 2022 White Paper listed important measures for expanding the country's electrical mix, enhancing service quality, and expanding access to energy. Establishing energy as a transformative good, positioning Kenya as a leader in decarbonized economic growth globally, installing 100 GW of renewable energy by 2040, and positioning Kenya as an investment destination for decarbonizing sectors. As a crucial component of its plan to use only renewable energy by 2030, Kenya is also actively investigating the possibilities of green hydrogen. In this regard, Germany and the European Bank for Investment have pledged to assist Kenya [41]. Given that Kenya inked an agreement with an Australian energy business to create green ammonia, the private sector is becoming more and more active in this regard [42].

However, vulnerable populations shouldn't have to pay the price for these policies. It is still essential to support affordable energy and provide equitable possibilities for communities that are at risk or impacted by the switch to renewable energy sources. In order to speed up economic diversification, countries such as South Africa that rely significantly on fossil fuels must develop a suitable legislative framework. Safety nets will be necessary for workers in high-risk sectors, including mining, to guarantee that their livelihoods are not interrupted [43]. Making Africa a more attractive place to invest is also cardinal. Creating more customized guarantees and insurance solutions can be of practical importance in realizing this. Investors may thus be shielded by these tools against project failures or default risks, including currency, payment, and political or regulatory concerns. Guarantees might, for instance, pay for debts, offer currency hedging services, or give political risk insurance that protects against the possibility of civil upheaval, war, or expropriation.

Through creative financing methods like securitization, blended finance, or public-private partnerships, the private sector can also use its knowledge of financial de-risking as in other regions of the world. By utilizing resources from the public and commercial sectors, sharing risks and rewards across stakeholders, and diversifying funding sources, these arrangements can help lower risks. For example, bonds backed by the cash flows from future renewable energy assets can be issued through securitization. To lower the capital costs of renewable energy projects, blended finance can integrate financing from the public and private sectors [44]. However, despite these steps, land access is frequently a major obstacle to entry for renewable energy projects, necessitating multi-level action. African governments can help increase the area suitable for the installation of renewables by promoting policies around alternate uses of lands, such as wastelands or agrivoltaics land (which can be utilized for agricultural and solar-PV energy generation) [43]. Faster development work completion may also be possible if project developers involve local communities early and consistently, especially by having conversations about how the project will benefit them. Additionally, cutting-edge solutions such as floating solar photovoltaics can help expand the installation of renewable energy projects and get around land limits [43].

Power market frameworks are also a viable alternative that can be created by policymakers to promote and draw in private sector investment. This ought to encourage competition, facilitate the creation of an effective electrical market, and incorporate international power trade. In Africa's transition to renewable energy, delivering in these areas can be crucial, particularly if regional harmonization of laws and regulations is also implemented. In order to overcome persistent obstacles that have impeded private sector involvement in Africa's electrical markets, an effective electricity market design is also necessary. The private sector may assist by exchanging knowledge and insights regarding challenges it has encountered both domestically and internationally.

African countries can increase their chances of success in creating and enforcing their suggested supporting renewable energy policies and regulations by using public-private partnership structures and other methods of organizing the private sector's involvement [43]. Examples include cutting red tape, simplifying planning and regulatory processes, and expediting the licensing and permitting process. A larger pool of dedicated and involved private sector businesses bidding for project development opportunities could result in a more competitive and dependable electricity supply, encourage the development of sustainable energy, and reduce electricity costs for businesses and consumers.

Since there aren't enough resources to sustain this delivery, the emphasis is frequently on areas that are thought to be more important than renewable energy. As a result, there is frequently a lack of political will and policy coherence with regard to coverage and length for the majority of areas. This combination leads to Africa's low adoption of renewable energy prospects in the energy sector [44]. Making renewable energy a priority and placing it high on the agenda will allow governments, private sector entities, and foreign development partners to work together more effectively to address these issues. There is a chance to successfully address the issues mentioned above if everyone participates and applies the necessary skills and expertise.

In achieving this, several initiatives have been put in place. Many African nations have significantly altered their legal and policy structures. Africa and Europe are currently working to enhance Africa's power industry through flagship projects including the Continental Power System Masterplan and the African Single Electricity Market. This has yielded useful lessons and insights that can be implemented as best practices. It is well known that creating advantageous policies and regulations is crucial in assisting to ensure market openness, attractiveness, and readiness for renewable energy opportunities, even though there is no one-size-fits-all solution that can address the wide range of issues African nations face. This illustrates how important it is to have clear policies and regulations.

As shown in Table 3, the total external debt stock primarily for Africa has a positive and significant effect on the Net ODA received by the continent while the interest payments on long term external debt, disbursement and IMF credit and SDR allocation all had a negative effect on the ODA to African countries. Firstly, the result on debt servicing is staggering and provides context to the matter of whether ODA should be used as instrument to re-orient a country's resources from debt servicing to economic production. If debt servicing negatively affects ODA inflows, then it makes sense to put ODA inflows pivotal to the discussions of external debt sustainability irrespective of distributional or utilization concerns that have undermined its role, particularly for African countries in the recent past.

**Table 3:** ODA and external debt.

Variable	(1)	(2)	(3)	(4)
Net ODA per capita (L1)	0.0012	0.3452∗	0.4374^a^	0.5072
	(0.0935)	(0.1739)	(0.0309)	(0.3262)
Bilateral	−2.5388	1.2752	−1.1963^a^	0.4778
	(1.8429)	(3.2138)	(0.3893)	(1.8679)
Multilateral	−2.5155	1.3377	−0.1855	0.4999
	(1.8376)	(3.2135)	(0.3867)	(1.8851)
Public sector	0.06781^a^	0.02545∗∗	0.0113	0.0118
	(0.0288)	(0.0124)	(0.0075)	(0.0262)
Private creditors	−0.0717^a^	−0.0134	−0.0201∗∗	−0.0317
	(0.0229)	(0.0165)	(0.0111)	(0.0334)
Total External Debt	−0.0119	0.0135	0.0091^a^	−0.0162
	(0.0116)	(0.0126)	(0.0023)	(0.0146)
Interest Payments	0.5429∗∗	−0.4095∗∗	−0.1808^a^	−0.1791
	(0.2129)	(0.1733)	(0.0679)	(0.2039)
Long Term External Debt	0.0201	−0.0115	0.0004	0.0404
	(0.0193)	(0.0193)	(0.004)	(0.032)
Official Creditors	2.4583	−1.3473	0.1668	−0.5292
	(1.8538)	(3.2119)	(0.3889)	(1.8868)
Constant	141.5156^a^	118.2148^a^	56.7469^a^	66.1684
	(35.64625)	(33.7425)	(10.7589)	(75.0432)
AR(1)	−0.41	−0.49	−1.45	−0.4
AR(2)	−1.16	0.52	−1.1	0.38
Sargan OIR	3.31	0.25	25.13	1.31
Hansen OIR	11.57	6.63	18.51	9.21
Instruments	21	21	21	21
Countries	39	39	39	39
Observations	390	390	390	390

a P < 0.001, ∗∗ = P < 0.05, ∗ = P < 0.1.

Indeed, as relative to exports and economic activity Africa's debt is the highest of any region in the world and thus its external debt burden reflects the inability to meet its debt service obligations in relation to its foreign currency earnings. Besides the negative effect of interest payments on long term external debt on ODA perhaps stems from the fact that an increase in interest payments reflects structural bottlenecks in the recipient country and thus provides an indication that aid may not necessarily be optimally utilized and thus a reduction in ODA which further explains the negative effect of the IMF credit. When ODA utilization indicators cannot be ascertained, debt service and disbarment can be employed to provide a picture a picture of the structural capabilities. Furthermore, this also extends to debt rescheduling by bilateral donors which in this case would not constitute true debt relief; rather it leads to a further buildup of the debt stock, as rescheduled debt often attracts higher interest rates which would increase the interest payments and become a source of capital flight pressures as many African countries are constrained to use approximately 20 percent of their export earnings for debt servicing.

### 5.4 Robustness analysis

#### 5.4.1 Sequential test for multiple breaks at unknown breakpoints

In a two-stage system GMM, determining structural change is an essential step in asserting the analysis's robustness. Since our investigation covered a longer period of time (2012–2022), there is a chance that significant disruptive events, such the COVID-19 outbreak, may have altered the model parameters. Therefore, identifying and dating breaks was important not only for estimating but also for comprehending change drivers and their impact on the linkages between JET, external debt, and ODA net inflows. In order to analyze potential multiple structural breaks in our system GMM, we subsequently employed the Sequential test for multiple breaks at unknown breakpoints.

As the estimation is designed under the null hypothesis of no structural change against the alternative of a specific number of changes/breaks, the test increases the number of breaks by one every time it is able to reject the null, starting at 0 breaks. Table 4 shows that the null hypothesis of 0 breaks is never rejected for the relationship between ODA and any other regressor which thus provides an indication or robustness. We therefore concluded that there are no breaks for the series employed at break point date.

**Table 4:** Ditzen, Karavias & Westerlund Sequential test for multiple breaks at unknown breakpoints.

Variables	F(1|0)	Bai & Perron crit (5 %)	Dates
ODA & Fossil Fuels	2.78	8.58	Non
ODA & Solar	0.78	8.58	Non
ODA & Renewable	0.11	8.58	Non
ODA & Total External debt stocks	0.31	8.58	Non
ODA & Electricity generation (TWh)	0.09	8.58	Non
ODA & Coal	0.06	8.58	Non
ODA & Gas	0.8	8.58	Non
ODA & Bioenergy	0.08	8.58	Non
ODA & Public sector	0.4	8.58	Non
ODA & Official creditors	2.12	8.58	Non
ODA & Private creditors	0.11	8.58	Non
ODA & Interest payments long term	0.01	8.58	Non
ODA & Long-term external debt	0.36	8.58	Non
ODA & Bilateral	1.22	8.58	Non
ODA & Multilateral	1.97	8.58	Non

## 6 Conclusion

The study concluded that in driving the JET, a key form of renewable energy for Africa that seems to possess the capacity to revamp the renewable energy sector in Africa is that of Solar. Despite this however, Africa's solar energy industry is still in its infancy compared to other countries as the continent's grid capacity is only 90 MW, and solar electricity is primarily focused in rural areas rather than cities [38]. The conclusion regarding the relationship between debt and ODA was that, if debt servicing has a negative impact on ODA inflows, it makes sense to prioritize ODA inflows in discussions concerning the sustainability of external debt, regardless of concerns about utilization that have recently weakened its importance, especially for Africa. Furthermore, the fact that an increase in interest payments indicates structural bottlenecks in the recipient nation and suggests that aid may not always be used to its full potential may also contribute to the detrimental impact of interest payments on long-term foreign debt on ODA.

Transferring food from places with surpluses to those with deficits is a crucial component of an integrated food policy that African governments should implement with regards to the bio energy sector. Together with better post-harvest storage, this will increase food security and lessen conflicts between the production of food and biofuel. Some African countries may need to enact land reform legislation and other checks and balances to ensure that large-scale bioenergy plantations do not evict local communities and indigenous peoples off their land. In Africa, more cooperation is needed at the regional, national, and local levels to make it easier to integrate contemporary bio-energy technology into energy systems. Effective institutional, legal, and regulatory frameworks addressing biomass production, conversion technologies, biodiversity, environmental concerns, and socioeconomic challenges can help achieve this. African countries should adopt legislation that prevents speculative purchases of agricultural property and guarantees fair and transparent land leases, building on the Committee on World Food Security's Voluntary Guidelines on the Responsible Governance of Tenure of property, Fisheries, and Forests.

## CRediT authorship contribution statement

**Kalimanshi Nsakaza:** Writing – review & editing, Writing – original draft, Visualization, Validation, Software, Resources, Project administration, Methodology, Investigation, Formal analysis, Data curation, Conceptualization. **Talumba Ireen Chilipaine:** Supervision.

## Data availability statement

The data will be available upon reasonable request through corresponding authors.

## Funding

No funding was received for this study.

## Declaration of competing interest

No potential conflict of interest was reported by the authors.

## Figures and Tables

**Table:** 

Angola	Eritrea	Liberia	Rwanda	Zambia
Cape Verde	Eswatini	Madagascar	Senegal	Zimbabwe
Central African Republic	Gabon	Malawi	Sierra Leone	
Chad	Gambia	Mali	Somalia	
Comoros	Ghana	Mauritania	South Africa	
Congo	Guinea	Mauritius	Sudan	
Cote d'Ivoire	Guinea-Bissau	Mozambique	Tanzania	
Democratic Republic of Congo	Kenya	Niger	Togo	
Djibouti	Lesotho	Nigeria	Uganda	
